# Phenolic compounds and vitamins in wild and cultivated apricot (*Prunus armeniaca* L.) fruits grown in irrigated and dry farming conditions

**DOI:** 10.1186/0717-6287-47-46

**Published:** 2014-09-23

**Authors:** Tuncay Kan, Muttalip Gundogdu, Sezai Ercisli, Ferhad Muradoglu, Ferit Celik, Mustafa Kenan Gecer, Ossama Kodad, Muhammad Zia-Ul-Haq

**Affiliations:** Agriculture Faculty, Horticulture Department, Inonu University, Malatya, Turkey; Agriculture Faculty, Biotechnology Department, Yuzuncu Yil University, Van, Turkey; Agriculture Faculty, Horticulture Department, Ataturk University, Erzurum, Turkey; Agriculture Faculty, Horticulture Department, Yuzuncu Yil University, Van, Turkey; Agriculture Faculty, Horticulture Department, Igdir University, Igdir, Turkey; Departement of Pomology, National School of Agriculture, Meknes, Morocco; The Patent Office Karachi, Karachi, Pakistan

**Keywords:** Apricot, Phytochemicals, Ripening, Irrigation, HPLC

## Abstract

**Background:**

Turkey is the main apricot producer in the world and apricots have been produced under both dry and irrigated conditions in the country. In this study, phenolic compounds and vitamins in fruits of one wild (Zerdali) and three main apricot cultivars (‘Cataloglu’, ‘Hacihaliloglu’ and ‘Kabaasi’) grown in both dry and irrigated conditions in Malatya provinces in Turkey were investigated.

**Results:**

The findings indicated that higher content of phenolic compounds and vitamins was found in apricot fruits grown in irrigated conditions. Among the cultivars, ‘Cataloglu’ had the highest rutin contents both in irrigated and dry farming conditions as 2855 μg in irrigated and 6952 μg per 100 g dried weight base in dry conditions and the highest chlorogenic acid content in irrigated and dry farming conditions were measured in fruits of ‘Hacıhaliloglu’ cultivar as 7542 μg and 15251 μg per 100 g dried weight base. Vitamin C contents in homogenates of fruit flesh and skin was found to be higher than β-caroten, retinol, vitamin E and lycopen contents in apricot fruits both in irrigated and dry farming conditions.

**Conclusion:**

The results suggested that apricot fruits grown in both dry and irrigated conditions had high health benefits phytochemicals and phytochemical content varied among cultivars and irrigation conditions as well. However, more detailed biological and pharmacological studies are needed for the demonstration and clarification of health benefits of apricot fruits.

## Background

Turkey is a leading producer of both fresh and dried apricots (*Prunus armeniaca* L.) in the world for a long time and the country is accepted second homeland of apricot after China [1]. Despite wide cultivation in many parts of the world, Malatya province located in eastern Turkey is particularly important for apricot production and processing due to its favourable climatic and geographical conditions and the province called center of apricot in the World.

Apricot fruits are rich in minerals, organic acids, phenolic compounds and carbohydrates [2–4]. The fruits can be consumed fresh or dried but most of the globally produced apricots are consumed as fresh. Moreover apricot kernels are utilized as raw materials in snack, pharmaceutical and in cosmetic industries [5, 6].

Phenolic compounds exist in fruits and vegetables at varying levels and they have a determinant role in taste formation and they contribute to astringency and bitterness in horticultural crops [7–9]. Anthocyanins are phenolic compounds that are responsible for colour formation of fruits and vegetables. Phenolic compounds have an important role in fruit juice processing industry since they increase the turbidity and sedimentation of drinks such as fruit juices and wines [10]. Phenolics, in particular, are thought to act as antioxidant, anti-carcinogenic, anti-microbial, anti-allergic, anti-mutagenic and anti-inflammatory, as well as reduce cardiovascular diseases [11].

The apricot fruits belongs to different cultivars contain different levels of polyphenols as summarized by Macheix et al. [12]. Chlorogenic acid (5-caffeoylquinic acid) is the dominant phenolic compound in apricots. The other phenolic compounds determined in apricots are neochlorogenic acid, caffeic acid, p-coumaric acid, ferulic acid and their esters. (+)-Catechin and (-)-epicatechin are also determined in apricot fruits and their products [13–15]. Flavonols in apricots occur mostly as glucosides and rutinosides of quercetin and of kaempferol, however, quercetin 3-rutinoside (rutin) predominates [13, 16]. Apricot fruits contain different levels of phytochemicals such as vitamins, carotenoids and polyphenols, which are the determinants of taste, colour and nutritive values of the fruits [17, 18].

The study aimed to investigate the phenolic compounds and vitamins in fruits of wild and cultivated apricots grown in dry and irrigated farming conditions. Rapidly increasing demand on world water resources brings pressure on use of irrigation water as well as use of drinking water. In these circumstances, the study is particularly important as its aims to investigate the effects of dry farming on some important human health related biochemical characteristics of apricots.

## Results and discussion

### Phenolic profile

The study reports the effects of irrigation practices on human health related biochemical contents of cultivated (cvs. ‘Cataloglu’ , ‘Hacihaliloglu’ , ‘Kabaasi)’ and wild (Zerdali) apricot genotype. The findings in general indicated remarkably lower contents of phenolic compounds in the fruits of apricots grown in irrigated conditions compared to their dry farmed counterparts.

In terms of phenolic compound profile, chlorogenic acid and rutin were found to be the predominant phenolics (Table 1). The highest epicatechin and gallic acid content in both irrigated and dry farming conditions was recorded in fruits of ‘Cataloglu’ apricot cultivar as 424 and 1654 μg; 130 and 282 μg per 100 g dry fruit base, respectively (Table 1). Chlorogenic acid contents of Hacihaliloglu apricot were measured as 7543 μg per 100 g dry fruit in irrigated farming conditions and as 15252 μg per 100 g in dry farming conditions (Table 1). Contents of *p*-coumaric acid, ferulic acid, procyanidin (B1, B2, B3), catechin, rutin, caffeic acid, epigallocatechin and 3-B-Q-D were generally found to be higher in dry farming conditions (Table 1). The analysis of phenolic compounds indicated that fruits of ‘Cataloglu’ apricot cultivar had the highest rutin, epicatechin, gallic acid, epigalloketechin and 3-B-Q-D; Hacihaliloglu apricot fruits had the highest chlorogenic acid and procyanidin B2 (dry-farming conditions), Kabaasi apricot fruits had the highest p-coumaric acid, ferulic acid (dry-farming conditions), procyanidin B1, catechin, procyanidin B2 (irrigated) and caffeic acid and wild apricot (Zerdali) fruits had the highest contents of ferulic (irrigated), 3-B-Q-D (dry-farming) and procyanidin contents (Table 1). In the study of Dragovic-Uzelac et al. [19], gallic acid contents of ‘Keckemetska’ apricots grown in Baranja region in Croatia were recorded as 3.47, 2.35 and 2.43 mg per kg in immature, semi-mature and full mature stages, respectively. Those authors also reported chlorogenic acid contents in the three maturity stages in apricot as 18.87, 16.05 and 14.69 mg per kg. They also investigated the changes in caffeic acid, p-coumaric acid, ferulic acid, catechin, epicatechin and procyanidin (B1,B2,B3) contents according to maturity stages. Sultana et al. [20], measured *p*-coumaric acid content in dry apricot fruit and reported as 23.6 mg per kg, ferulic acid content as 13.9 mg per kg, caffeic acid content as 6.70 mg per kg and gallic acid content as 4.54 mg per kg. Similar studies have been conducted also by other researchers [13, 21]. While some of the findings of this study conducted in Malatya region are in accord with the findings of other researchers, some findings are in discord with those findings. These differences may be attributed to the differing characteristics of cultivars used in studies as well as differences in crop procedures, climatic conditions and geographical factors. Flavonoid glycosides, a group of phenolic compounds, are light yellow in colour and found in almost all plants. As their synthesis in plants is stimulated by sunlight, they are largely found in fruit skins. Flavonoid glycosides are major determinants of colour formation, hence, climatic factors such as temperature and light are considered to have an important role [10].

**Table 1:** **Phenolic compounds contents in standard apricot cultivars fruits grown in irrigated and dry farming conditions (**μ**g 100 g** ^**-1**^ **dw)**

Phenolıc compounds	İrrigated/Dry	Cultivars			
		Cataloglu	Hacıhaliloglu	Kabaasi	Zerdali
Rutin	Irrigated	2855.10 ± 0.14^a*^	1550.25 ± 0.17^d^	2656.10 ± 0.19^b^	1760.90 ± 0.26^c^
	Dry	6952.95 ± 0.76^a^	3946.90 ± 0.29^d^	6642.70 ± 0.59^b^	4159.60 ± 0.32^c^
Epicatechin	Irrigated	423.55 ± 0.11^a^	40.40 ± 0.20^d^	310.70 ± 0.60^c^	365.40 ± 0.20^b^
	Dry	1653.60 ± 0.98^a^	156.30 ± 0.11^d^	657.65 ± 0.33^c^	871.85 ± 0.24^b^
P-coumaric	Irrigated	5.40 ± 0.30^b^	7.50 ± 0.40^b^	21.55 ± 0.11^a^	5.30 ± 0.30^b^
	Dry	15.50 ± 0.30^c^	32.60 ± 0.11^b^	44.00 ± 0.21^a^	13.05 ± 0.15^c^
Ferulic	Irrigated	431.25 ± 0.12^c^	372.75 ± 0.67^d^	884.75 ± 0.10^b^	1136.90 ± 0.16^a^
	Dry	1057.05 ± 0.28^c^	1044.05 ± 0.20^c^	2440.75 ± 0.25^a^	1446.35 ± 0.95^b^
Gallic acid	Irrigated	129.75 ± 0.81^a^	87.35 ± 0.36^b^	35.40 ± 0.13^c^	49.30 ± 0.40^c^
	Dry	282.30 ± 0.60^a^	216.40 ± 0.51^b^	75.15 ± 0.12^d^	108.95 ± 0.56^c^
Procyanidin B1	Irrigated	46.90 ± 0.23^d^	72.55 ± 0.43^bc^	82.85 ± 0.19^a^	63.80 ± 0.13^c^
	Dry	112.10 ± 0.35^c^	157.30 ± 0.76^b^	236.15 ± 0.18^a^	77.45 ± 0.21^c^
Catechin	Irrigated	1361.95 ± 0.37^c^	1560.85 ± 0.31^b^	2140.60 ± 0.77^a^	1067.65 ± 0.14^d^
	Dry	2746.20 ± 0.11^c^	3580.30 ± 0.17^b^	5124.95 ± 0.21^a^	2210.25 ± 0.21^d^
Procyanidin B2	Irrigated	654.20 ± 0.19^d^	7140.50 ± 0.28^b^	7792.70 ± 0.40^a^	6740.40 ± 0.16^c^
	Dry	1552.40 ± 0.32^d^	19534.20 ± 0.97^a^	16563.65 ± 0.47^b^	15405.55 ± 0.15^c^
Caffeic acid	Irrigated	62.85 ± 0.25^d^	187.10 ± 0.82^c^	359.35 ± 0.13^a^	276.95 ± 0.19^b^
	Dry	173.95 ± 0.16^c^	646.80 ± 0.31^b^	845.20 ± 0.23^a^	638.00 ± 0.16^b^
Epigallocatechin	Irrigated	229.25 ± 0.73^a^	34.41 ± 0.23^c^	52.80 ± 0.19^b^	64.05 ± 0.15^b^
	Dry	557.55 ± 0.22^a^	96.60 ± 0.31^b^	134.00 ± 0.19^b^	141.60 ± 0.59^b^
3-B-Q-D	Irrigated	271.95 ± 0.14^a^	29.85 ± 0.11^c^	26.60 ± 0.10^c^	192.70 ± 0.44^b^
	Dry	446.80 ± 0.28^a^	106.45 ± 0.46^b^	65.20 ± 0.33^b^	405.35 ± 0.45^a^
Procyanidin B3	Irrigated	561.00 ± 0.61^c^	729.30 ± 0.78^b^	727.15 ± 0.16^b^	1641.20 ± 0.13^a^
	Dry	2129.75 ± 0.15^b^	1652.40 ± 0.13^c^	1657.85 ± 0.35^c^	2960.95 ± 0.27^a^
Chlorogenic acid	Irrigated	3648.65 ± 0.23^b^	7542.75 ± 0.58^a^	585.00 ± 0.10^d^	1061.80 ± 0.23^c^
	Dry	6551.15 ± 0.65^b^	15251.50 ± 0.15^a^	1345.95 ± 0.13^d^	2634.50 ± 0.30^c^

*Different letters in lines indicate significantly different values at p ≤ 0.05.

### Vitamin content

The effects of irrigated and dry farming conditions on β-carotene, retinol, vitamin E, lycopen and vitamin C contents in fruits of examined apricots were investigated. The highest β-carotene contents in both irrigated and dry farming conditions were measured in fruits of ‘Hacıhaliloglu’ apricot cultivar as 1949 μg per 100 g and 3275 μg per 100 g (Table 2). The highest vitamin C content in both irrigated and dry farming conditions was measured in fruits of ‘Cataloglu’ cultivar as 20246 μg and 60656 μg per 100 g dry weight base (Table 2). The highest retinol content in irrigated farming conditions was measured in fruits of ‘Hacıhaliloglu’ cultivar as 21.15 μg per 100 g dry weight base and the highest retinol content in dry farming conditions was measured in fruits of ‘Cataloglu’ apricot cultivar as 28.10 μg per 100 g. In general, the ranking of vitamin content in the examined apricots was determined as Vitamin C > βeta caroten > Lycopen > Vitamin E > Retinol (Table 2). Dragovic-Uzelac et al. [19] conducted a study on ‘Keckemetska Ruza’ , ‘Madjarska Najbolja’ and ‘Velika Rana’ apricot cultivars grown in Neretva valley and they determined β-caroten contents of these apricot fruits in ripeness stage as 796, 1375, and 948 μg per 100 g, respectively. Rigal et al. [22] measured the lycopene contents in control apricots and dry apricots respectively as 3.2 μg and 3.1 μg per g. Akin et al. [23] determined β-carotene contents as 8.88 mg per 100 g in fruits of ‘Hacıhaliloglu’ , 21.59 mg per 100 g in fruits of cv. ‘Tokaloglu’ and 26.18 mg per 100 g in fruits of cv. ‘Kabaasi’ apricots, respectively. Same researchers measured vitamin C contents as 37.7, 41.6 and 27.9 mg per 100 g in fruits of ‘Hacıhaliloglu’ , ‘Kabaasi’ and ‘Cataloglu’ apricot cultivars, respectively. The findings of this study are partially in accord with the findings of other researchers and comparatively higher and lower findings are also observed in this study. Vitamin C is highly affected by factors such as temperature, light etc. and degrades fastly [10]. Therefore, the differences in vitamin contents of examined cultivars are attributed to cultivar characteristics, applied crop procedures and environmental factors.

**Table 2:** **Vitamins compounds contents in standard apricot cultivars fruits grown in irrigated and dry farming conditions (**μ**g per 100 g dw)**

Vitamin compounds	İrrigated/Dry	Cultivars			
		Cataloglu	Hacıhaliloglu	Kabaasi	Zerdali
Betacaroten	Irrigated	1150.85 ± 0.85^b*^	1949.25 ± 0.28^a^	1176.55 ± 0.21^b^	532.60 ± 0.39^c^
	Dry	1567.80 ± 0.17^c^	3274.85 ± 0.20^a^	2675.75 ± 0.17^b^	1338.90 ± 0.16^d^
Retinol	Irrigated	18.15 ± 0.55^b^	21.15 ± 0.35^a^	13.10 ± 0.70^c^	10.95 ± 0.13^d^
	Dry	28.10 ± 0.50^a^	23.95 ± 0.35^b^	21.35 ± 0.45^c^	21.95 ± 0.45^c^
Vitamin E	Irrigated	37.60 ± 0.40^a^	38.30 ± 0.35^a^	27.70 ± 0.80^b^	27.80 ± 0.80^b^
	Dry	84.25 ± 0.75^a^	85.10 ± 0.90^a^	61.90 ± 0.16^b^	62.40 ± 0.13^b^
Lycopen	Irrigated	57.85 ± 0.94^b^	71.05 ± 0.45^a^	47.80 ± 0.20^c^	66.10 ± 0.19^a^
	Dry	283.60 ± 0.89^a^	186.30 ± 0.60^c^	246.55 ± 0.48^b^	154.95 ± 0.80^d^
Vitamin C	Irrigated	20245.95 ± 0.41^a^	7341.05 ± 0.13^d^	11547.55 ± 0.36^b^	8732.55 ± 0.41^c^
	Dry	60655.85 ± 0.41^a^	13549.35 ± 0.28^d^	18660.90 ± 0.82^c^	26148.20 ± 0.37^b^

*Different letters in lines indicate significantly different values at p ≤ 0.05.

## Conclusion

Turkey is the world’s leading apricot producer and Malatya province is the main apricot growing center in Turkey. The findings of the study indicated that higher contents of phenolic compounds and vitamins are found in fruits grown dry-farming conditions compared to their irrigated counterparts. Phenolic compounds may cause some problems in fruit juice processing industry as they increase turbidity. Hence, irrigated farming conditions may be advantageous in this respect as they result lower contents of phenolics in cultivars. Phenolic compounds and vitamins are important for human health due to their antioxidant properties. Dry-farming conditions are more advantageous in this sense as they result higher contents of phenolics and vitamins in cultivars. Many studies have been conducted on biochemical contents of apricot fruits. Nevertheless, there is limited research investigating the effect of irrigation on biochemical contents of apricots. The study will contribute to literature in this regard and is thought to be a valuable reference for the forthcoming studies.

## Methods

### Plant material

In the study 3 main commercial apricot cultivars (‘Cataloglu’ , ‘Hacihaliloglu’ and ‘Kabaasi’) and 1 wild apricot (Zerdali) genotype both grown in same dry and irrigated farming orchards were used. The trees of cultivars were grafted on seedlings of wild apricots. However wild apricot (Zerdali) was ungrafted that grown on their roots. All trees were 11 years old and found 1050 meter a.s.l. with 38°49′N, 37°56′E position. The study was conducted in Hekimhan district belongs to Malatya province which is the main apricot growing center in Turkey with its convenient climatic and geographic features. All cultivars and wild material were planted at 5×4 m row spacing and intra-row spacing in single crop orchards. Drip irrigation with 15 days intervals was employed for irrigated farming conditions. In dry farming conditions, no irrigation was performed and the examined cultivars only utilized rain water. A total 50 fruits of cultivars and wild material were harvested at commercial maturity period (fully orange-ripe stage) and quickly transferred to the laboratory. Then fruits were cut into small pieces, frozen in liquid nitrogen and stored at -80°C for subsequent analysis.

### Extraction and determination of phenolic acids and flavonoids

The polyphenols in examined samples were extracted using a procedure described by Bengoecha et al. [24] Each apricot fruit puree (50 g) was mixed with 50 mL methanol/HCl (100:1, v/v) which contained 2% *t*BHQ, in inert atmosphere (N ) during 14 h at 35°C in dark. The extract was then centrifuged at 5000 rpm 15 min. Supernatant was evaporated to dryness under reduced pressure (35-40°C). The residue was dissolved in 25 mL of water/ethanol (80:20, v/v) and extracted four times with 25 mL of ethyl acetate. The organic fractions were combined, dried for 30-45 min with anhydrous sodium sulphate, filtered through the Whatman No. 40 filter paper (Whatman International Ltd., Kent, England) and evaporated to dryness under vacuum (35-40°C). The residue was dissolved in 1 mL of methanol/water (50:50, v/v) and filtered through 0.45 μm filter (Nylon Membranes, Supelco Inc., Bellefonte, PA, USA) before injected (20 μL) into the HPLC apparatus. Samples were extracted in triplicate.

Extractions were performed on a DionexASE 200 (Dionex Corp., Sunnyvale, CA, USA) system. Percentages of methanol and water in the solvent, temperature, pressure and static extraction time were the parameters under study. The pre-set default conditions were as follows: pre-heating period, 5 min; solvent flush volume, 6 0% of the extraction cell volume; number of extraction cycles, 3; purge, 90 s using pressurized nitrogen (99.995% of purity, 150 p.s.i.); and collection, in 60 ml glass vials with teflon coated rubber caps (I-CHEM,New Castle, DE, USA). The solvent used was previously degassed in order to avoid the oxidation of the analytes under the operating conditions. Optimum extraction conditions were determined as 1500 psi pressure, 40°C temperatures and an hour application time in PLE system. PEE (1 g) was mixed in gradient conditions with (methanol/water/hydrochloric acid; 75:20:5) which contained 2% tBHQ in 11-or 22-ml stainless steelextraction cells. Then, each one was filtered through a 0.45 μm nylon membrane (Lida, Kenosha, WI, USA) and transferred to a 50 ml volumetric flask. Solvents were evaporated to dryness in a Turbovap LV Evaporator (Zymark, Hopkinton, MA, USA) provided with a nitrogen stream in a water bath at 40°C. The residue was reconstituted in (2 ml) methanol-water-aqueous (50:50, v/v) and which was brought up to its volume with methanol-water filtered through a 0.45 μm PTFE filters (Waters, Milford, CA, USA) prior to injection into the HPLC system.

Polyphenol analysis were performed on a Agilent Series 1100 liquid chromatography, equipped with a vacuum degasser, a quaternary pump and a Agilent 1100, G 1315B DAD detector, connected to a HPChemStation software. A reversed-phase ACE 5 C18A11608 (250×4.6 mm, 4 μm) column was used. The content of solvents and used gradient elution conditions were previously described by Dragovic-Uzelac et al. [15]. For gradient elution mobile phase a contained 3% acetic acid in water; solution B contained a mixture of 3% acetic acid, 25% acetonitrile and 72% water. The following gradient was used: 0-40 min, from 100% A to 30% A, 70% B with flow rate 1 mL/min; 40-45 min, from 30% A, 70% B to 20%A, 80% B with flow rate 1 mL/min; 45-55 min, from 20% A, 80% B to 15% A, 85% B with flow rate 1.2 mL/min; 55-57 min, from 15% A, 85% B to 10% A, 90% B with flowrate 1.2 mL/min; and 57-75 min 10% A, 90% B with flow rate 1.2 mL/min. Operating conditions were as follows: column temperature, 30°C, injection volume, 20 μL, UV-VIS photo diode array detection at 280 nm. Detection was performed with UV-VIS photo diode array detector by scanning spectra from 210 to 360 nm. Stock standard solutions at a concentration of 1 mg ml, G1 were prepared in methanol/water (1:1) and stored at 4°C in darkness. Different range calibration curves for different polyphenol components in apricot samples were used.

### Analytical quality control

Recoveries were measured by comparing retention times and spectral data with those of authentic standards. Quantitative determinations were carried out using calibration curves of the standards.

### Extraction and determination of vitamins

Apricot samples (50 g) were mashed in a homogenizer and 4 g homogenate paste per sample were taken for extraction of vitamins A, E, β-carotene, lycopen and 1 g per sample was taken for extraction of vitamin C. To the above homogenates, 4 ml of ethanol were added, vortexed and the mixture centrifuged (Mistral#2000) at 2000 rpm for 3 min at 4 h. The supernatant was also filtered through a Whatman paper, and to the filtrate 0.15 ml n-hexane was added and mixed. Vitamins A, E and β-carotene were extracted twice in the hexane phase and the collected extract was dried under a stream of liquid nitrogen. Dried extract was solubilized in 0.2 ml methanol for HPLC. Injections were made in duplicate for each sample. The quantification [25] utilized absorption spectra of 326, 296 and 436 nm for vitamins A, E, and b-carotene, respectively.

HPLC separations were accomplished at room temperature consisting of a sample injection valve (Cotati 7125) with a 20 μl sample loop, an ultraviolet (UV) spectrophotometric detector (and a Techsphere ODS-2 packed (5 mm particle and 80 A pore size) column (250_4.6 i.d.) with a methanol: acetonitrile: chloroform (47:42:11, v/v) mobile phase at 1 ml per min flow rate. The extraction of vitamin C was according to the method of Cerhata et al. [26]. To 1 g homogenized apricot paste, 1 ml of 0.5 M perchloric acid was added, vortexed and the volume adjusted to 5 ml by adding ddH2O. The mixture was centrifuged at 5000 rpm for 8 min at 4°C, the supernatant was filtered, as earlier, and the vitamin C level was determined using the method of Tavazzi et al. [27] by HPLC, utilizing a column (250_3.9 i.d.) packed with Tecopak C18 reversed-phase material (10 mm particle size) with mobile phase (3.7 mM phosphate buffer, pH 4.0) at 1 ml min 1 flow rate.

### Statistical analysis

Experimental data were evaluated using analysis of variance (ANOVA) and significant differences among the means of three replicates (*p* < 0.05) were determined by Duncan’s multiple range test, using the “SPSS 10.0 for Windows”.
